# Study on Miscibility, Thermomechanical Behavior, and Thermoregulation Performance of Paraffin Wax/Bituminous Blends for Solar Thermal Energy Storage Applications

**DOI:** 10.1021/acs.energyfuels.3c04229

**Published:** 2024-01-26

**Authors:** Coraima Gutiérrez-Blandón, Antonio A. Cuadri, Clara Delgado-Sánchez, Pedro Partal, Francisco J. Navarro

**Affiliations:** Pro^2^TecS-Chemical Process and Product Technology Research Centre, Department of Chemical Engineering, ETSI, Campus de “El Carmen”, Universidad de Huelva, 21071 Huelva, Spain

## Abstract

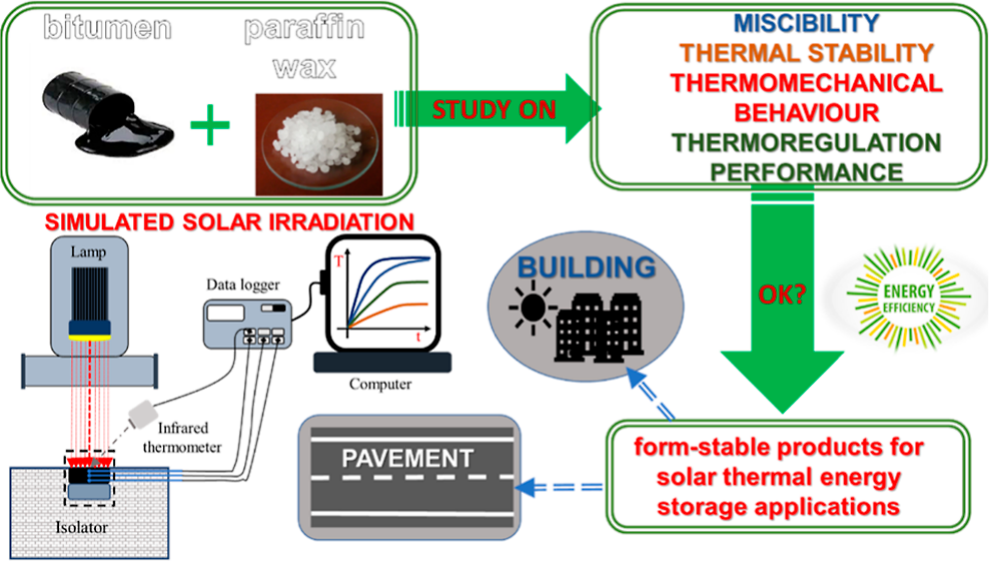

The goal of this work was to study the miscibility, thermal stability, thermomechanical properties, and temperature regulation performance of paraffin wax/bitumen blends for their potential use in solar thermal energy storage applications. Results indicated that these blends present a suitable thermal stability, and their thermomechanical properties are strongly dependent on composition, developed microstructure, and temperature. Among all paraffin wax concentrations studied, the blend containing 40 wt % paraffin wax displays enhanced binder elastic properties together with lower thermal susceptibility compared to base bitumen. In addition, this binder also presents improved thermal properties (thermal conductivity and specific heat capacity) and still maintains a high crystallinity, thereby retaining a large enough latent heat to be used for thermal energy storage. Thus, results from the temperature regulation test, which was conducted by subjecting the sample to simulated solar irradiation at a constant radiant flux density, provide a higher latent heat thermoregulation index value than other microencapsulated phase change materials systems. Therefore, it can be stated that paraffin wax/bitumen blends are promising base materials to formulate form-stable products for thermal energy storage applications for thermoregulation purposes.

## 1 Introduction

Bitumen, a complex mixture of organic molecules from crude oil distillation, presents suitable properties (superior waterproof, adhesive properties, low in cost, etc.) to be used in civil engineering, with long-established uses. The most widespread application of bitumen is as a binder for mineral aggregates in roads pavements, but it also has been widely used in roofing and waterproofing membranes for building applications.^1−4^

The significant increase in the price and consumption of energy is forcing industry and public administration to develop new design strategies aimed at obtaining energy-efficient products. As an example, the building sector, which is responsible for over one-third of the overall energy demand worldwide, is expected to reduce its environmental footprint to avert the expected 50% rise in energy demand before 2050.^5^ Consequently, the petrochemical industry is encouraged to develop new energy efficient technologies of thermal energy storage by using novel, low cost, and effective bituminous materials. On these grounds, the use of solid-to-liquid phase change materials (PCMs), which are able to store/release large amounts of thermal latent energy during the melting/crystallization processes in a narrow temperature interval, has lately gathered increasing attention by the scientific community.^6−9^ Among them, organic paraffin waxes are preferred as PCMs because of some unique features such as large energy density, low supercooling, good chemical and phase change stability, low in cost, etc.^10^

One of the main technical issues is how to effectively integrate the PCM within the supporting engineering material, such as bitumen, to prevent leakage and volatilization.^11^ To that end, the encapsulation of PCMs is considered the most mature technology to manufacture PCM/bituminous materials with advanced thermal functionalities for pavement and building applications. Thus, bitumen doped with adequate encapsulated PCMs is effective in dampening changes in pavement temperatures and, thereby, can contribute to minimizing the pavement failures associated with the extreme in-service temperatures, such as rutting and cracking.^12−14^ In addition, encapsulated PCMs have also been employed to manufacture road pavement solar collectors.^10,15^ As for building applications, encapsulated PCMs act as thermo-regulating agents, contributing to thermal comfort and energy saving.^12,16^ However, this strategy presents some disadvantages that would restrict its industrial application: (i) a substantial increase in the final price, (ii) a reduction in the efficiency of the heat-transfer processes due to the low thermal conductivity of the compounds used to create the shell, and (iii) a reduction in the effective concentration of PCMs in the final product.^12,17^ Interestingly, form-stable PCM/bituminous materials are one of the strategies currently being used for encapsulation. This technology consists of the creation of a polymer/bitumen matrix with enough global stiffness to fix the PCM, preventing its leakage during the melting process.^18−20^ Thus, the use of form-stable PCM/bituminous materials would be a cheaper method to obtain energy-efficient bituminous products with advanced thermal properties for paving and building applications. However, the lack of knowledge about the miscibility between a complex material such as bitumen and the selected PCM limits the development of these multiphasic materials on an industrial scale. In this sense, for an adequate implementation of this strategy, a partial miscibility between bitumen and PCM molecules is required to achieve a stable dispersion (which would not be possible for a very low miscibility) without losing the latent heat storage capacity (which would occur if both components are fully miscible).^4,21−23^

Therefore, considering the excellent properties of bitumen as supporting materials in paving and building applications, as well as the unique features exhibited by organic paraffin waxes as PCMs, the main objective of this work was to explore the miscibility, thermal stability, and thermomechanical behavior of paraffin wax/bitumen blends for solar energy storage applications. Their applicability was evaluated by means of rheological measurements, differential scanning calorimetry (DSC), thermogravimetric analysis (TGA), technological properties, and chemical composition analysis. After that, the thermal conductivity and specific heat capacity were determined, and the temperature regulation performance of a selected paraffin wax/bituminous blend was evaluated using simulated solar irradiation. The results revealed that paraffin wax/bitumen blends are promising base materials to formulate form-stable products for solar thermal energy storage applications for thermoregulation purposes.

## 2 Experimental Section

### 2.1 Materials

A paraffin wax (referred to as P), supplied by Panreac-AppliChem (Spain), with a melting temperature around 60 °C was selected as the PCM. A bitumen (referred to as B) with penetration grade within the range 100/150, donated by REPSOL S.A. (Spain), was used as a supporting engineering material for the manufacture of the PCM-bituminous blends. This bitumen has a ring-and-ball softening point of 41.0 °C and a penetration value of 105 dmm, according to UNE-EN 1427 and 1426 standard, respectively. Its chemical composition (in terms of its SARAs fraction) is of 7.4 wt % saturates, 57.6 wt % aromatics, 15.1 wt % resins, and 19.9 wt % asphaltenes.

### 2.2 Sample Preparation

Blends of bitumen with 2, 5, 20, and 40 wt % paraffin wax were mixed for 15 min in a cylindrical vessel at 150 °C and an agitation speed of 3500 rpm by using a rotor-stator homogenizer (Silverson L5M-A). Previously, both compounds were tempered at the processing temperature for 1 h. After blending, the resulting material was divided into small containers and allowed to cool to ambient temperature. With regard to their nomenclature, a bitumen/paraffin wax blend containing 2 wt % paraffin will be labeled as BP2 and so on.

### 2.3 Tests and Measurements

#### 2.3.1 Rheological Characterization

Oscillatory-shear temperature sweep tests, from 30 °C up to the maximum possible temperature for reliability in the measurements, were carried out in a controlled-stress Physica MCR-301 rheometer (Anton Paar, Austria). Tests were conducted at a frequency of 10 rad/s, a heating rate of 1 °C/min, and by selecting strains so as to ensure a linear viscoelastic response within the whole testing temperature range. A smooth plate-and-plate geometry of 25 mm in diameter with a 1–2 mm gap was used.

For the sake of reproducibility, samples were submitted to the same preparation protocol. Thus, they were heated to 100 °C in an oven for thermal stabilization, poured into cylindrical silicone molds and, let slowly cool down to room temperature, and, finally, stored in a freezer at −20 °C before testing. This protocol allows the preservation of the microstructure until tests are subsequently conducted. Finally, after the samples were placed in the rheometer measuring system, they were equilibrated for at least 30 min at the testing temperature.

#### 2.3.2 Thermogravimetric Analysis Test

TGA tests were conducted in a TA Q-50 instrument (TA Instruments, USA). Temperature sweeps (10 °C·min^–1^; from 30 to 550 °C) were carried out on 5–10 mg samples of pure components and bituminous blends under an inert nitrogen atmosphere.

#### 2.3.3 Thin-Layer Chromatography with Flame Ionization Detector

Chemical composition, in terms of “SARAs” fraction (i.e., saturates, aromatics, resins, and asphaltenes), for bituminous binders was determined by means of thin-layer chromatography coupled with a flame ionization detector (TLC/FID), using an Iatroscan MK-6 analyzer (Iatron Corporation Inc., Japan). Elution was performed with heptane, toluene/heptane (80/20, in volume), and trichloroethylene/methanol (95/5, in volume), following the procedure outlined elsewhere.^24^

#### 2.3.4 Ring-and-ball Softening Temperature

Ring-and-ball softening temperature, a technological test typically used for bitumen characterization, refers to the temperature at which a steel ball deforms the binder contained in a metal ring under the specified testing conditions stated in the UNE-EN 1427 standard. In this test, the bituminous sample is taken in two brass rings, and steel balls are placed on it. Then, the system is placed in a water bath and heated. The temperature at which a steel ball with a bitumen coating hits a surface located at a specific distance from the ring is called the ring-and-ball softening temperature.

#### 2.3.5 Differential Scanning Calorimetry Analysis and Specific Heat Capacity

DSC was performed with a Q-250 DSC (TA Instruments, USA). Tests were carried out under a N_2_ atmosphere at a flow rate of 50 mL min^–1^, with a heating/cooling rates of 3 °C min^–1^, using 10–20 mg samples sealed in hermetic aluminum pans. In order to ensure the same recent thermal history, samples were heated to 120 °C for 10 min and then subjected to the cooling ramp to −80 °C, kept at this temperature for 10 min to reach the thermal equilibrium, and, next, to the heating cycle to 120 °C. This pattern was repeated 50 times to measure the thermal reliability of samples.

The specific heat capacity was measured by modulated DSC, using a 2 °C min^–1^ heating ramp and a temperature modulation of ±0.3 °C for 60 s.

#### 2.3.6 Thermal Conductivity Measurement

The thermal conductivity, at different temperatures, was measured using the nondestructive Transient Hot-Bridge (THB) technique by a THB 100 device from Linseis GmbH (Germany). A sensor type A with a metal frame (A-13890) was used for the measurements. The sensor was placed between two equal flat faces of two samples (minimum sample size, 20 × 40 × 5 mm) of the same formulation and thermostated in a lab Heratherm oven (Thermo Scientific, Germany). Ten replicas were recorded for each sample and temperature.

#### 2.3.7 Temperature Regulation Test Using Simulated Solar Irradiation

**Figure 1:**
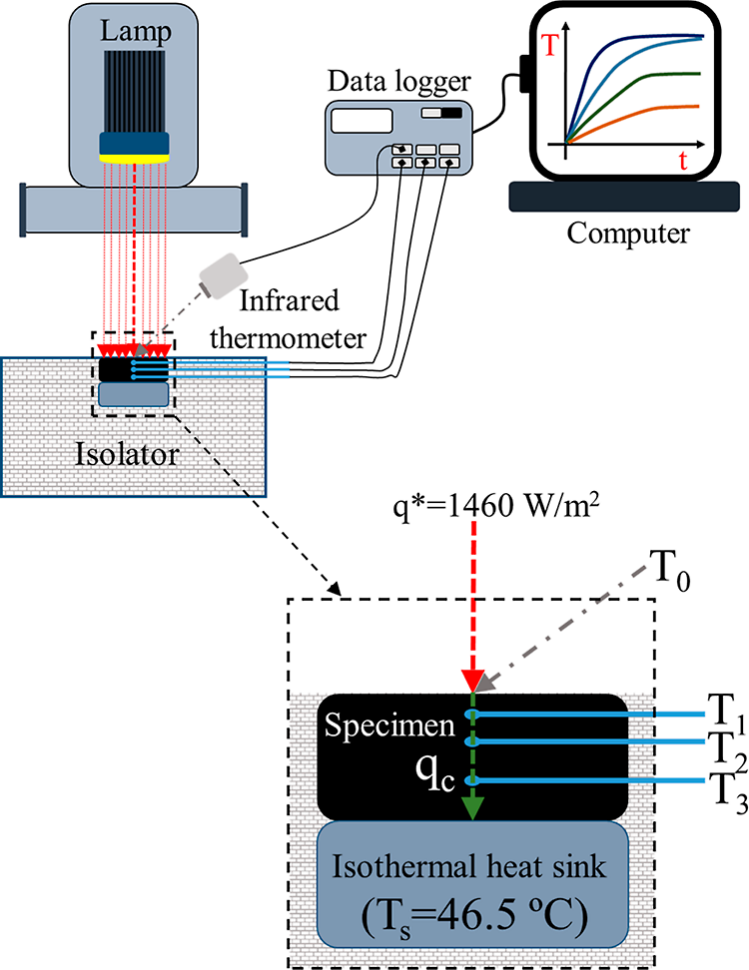
Scheme of the experimental setup used for the regulation temperature tests.

The temperature regulation tests were carried out by using a xenon lamp HXF300-T3 (Beijing China Education Au-light Technology Co., Ltd., China) with an attached filter AM 1.5G (300–1100 nm). Testing specimens consisted of a binder disk (42.0 mm diameter and 8.5 mm thickness) with four temperature sensors, three of them located at the center with readings at 2.2, 3.4, and 6.8 mm depth. These temperatures will be referred to as T_1_, T_2_, and T_3_, respectively, and were taken with Pt-100 temperature sensors (0.8 mm diameter, and an accuracy of ±0.15 °C). In addition, sample top surface temperature (referred to as T_0_) was measured with an infrared thermometer (accuracy ±0.5 °C) using a calculated material emissivity of 0.94 and 0.90 for the BP40 blend and base bitumen, respectively. The sample side wall was isolated with a 77.6 mm-thickness isolator (with a thermal conductivity of 0.044 W/m °C), whereas its bottom side was in contact with an isothermal heat sink with a selected cooling or sink temperature of 46.5 °C (labeled as T_S_). A pyranometer SMP3 (Kipp & Zonen, Netherlands) was used to measure the incident radiation, which was fixed at 1460 W/m^2^ (referred to as q*) at the sample center. Pt-100 temperature sensors and an infrared thermometer were connected to a DataTaker DT80-AL (Thermo Fisher Scientific Australia Pty Ltd.) data logging instrument for temperature data recorder every 1 s, which was in turn connected to the computer via USB port. All tests were conducted at a room temperature of 21 ± 0.5 °C. A scheme of this experimental setup is sketched in Figure 1.

## 3 Results and Discussion

### 3.1 Miscibility, Thermal Stability, and Rheological Properties

**Figure 2:**
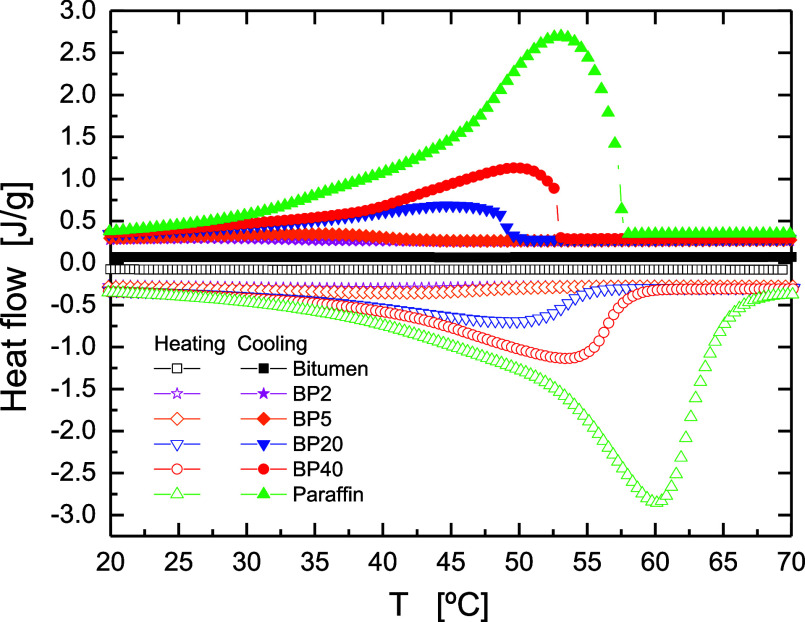
DSC heating and cooling scans of neat bitumen, paraffin wax, and their blends.

The development of form-stable paraffin wax/bituminous blends for thermal energy applications requires deep knowledge about the miscibility between paraffin wax and bitumen, as well as its effects on the crystallinity degree. In this sense, if both components are fully miscible, the latent heat storage capacity (or crystallinity) would be lost and, with that, their potential thermal energy storage applications; however, if the degree of miscibility is very low, macrophase separation happens, and then stable dispersions of both components cannot be achieved either. To that end, first, DSC tests were carried out on pure paraffin wax and its corresponding bituminous blends. All DSC scans (Figure 2) display wide asymmetric endothermic and exothermic events associated with the melting and crystallization of the crystalline structures, with maximum values (*T*_m_, *T*_c_) gathered in Table 1.

**Table 1:** Melting and Crystallization Temperatures (*T*_m_, *T*_c_), Crystallinity Degree, and Phase Change Enthalpy (Δ*H*_f_) for Pure Paraffin Wax and its Corresponding Paraffin Wax/Bituminous Blends

	*T*_m_ (°C)	*T*_c_ (°C)	crystallinity (%)	Δ*H*_f_ (J/g blend)	Δ*H*_f_ (J/g paraffin)
BP2	42.3	26.7	57.3	3.35	167.5
BP5	45.8	33.6	63.6	9.31	186.2
BP20	51.3	44.7	67.2	39.4	197.0
BP40	54.9	49.7	68.8	80.68	201.7
P	60.4	52.9	71.3	209.0	209.0

As may be seen, while for pure paraffin wax peak temperatures are centered at 60.4 and 52.9 °C, respectively, they are shifted to lower temperatures as paraffin wax concentration decreases, a result that hints a reduction in the quality and dimension of the crystals. In fact, Table 1 also points out a slight crystallinity decrease as paraffin concentration lowers. These outcomes may be explained by the fact that some crystallizable maltenic molecules, most probably waxes and saturates, naturally present in bitumen, could diffuse to the paraffin-rich phase, leading to modifications in the crystalline phase of paraffin wax.^25^ Anyway, the reported melting temperatures in Table 1 are within the specific temperature range for solar energy applications (from 40 to 80 °C).^26^

Crystallinity degree included in Table 1 has been calculated as follows
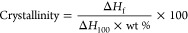
where Δ*H*_f_ is the phase change enthalpy of the blend, Δ*H*_100_ is the phase change enthalpy of perfectly crystalline polyethylene (293 J/g),^27^ and “wt %” represents the paraffin wax weight fraction in the blend. Therefore, on the one hand, results derived from DSC point out a partial miscibility between paraffin wax and bitumen. However, on the other hand, given the large retained crystallinity, the paraffin phase partially maintains its own identity in the blend and probably forms independent domains at a microscale level. Interestingly, despite the wide paraffin concentration range considered, the crystallinity degree is not reduced in a large extent, especially for high paraffin content (see BP40 in Table 1). Thus, the values of phase change enthalpy per gram of paraffin wax (Table 1) show a slight decrease compared to pristine paraffin wax, which is clearly ascribed to the minor reduction in crystallinity calculated for the blends. This finding is considered of great interest for the use of these materials for thermal energy storage applications, since a high fraction of crystallinity (and thereby a high phase change enthalpy) means a large capacity to store thermal energy. Thus, it is important to highlight that the specific melting enthalpies of the blends having large paraffin contents are similar to those reported in the bibliography for other form-stable PCMs.^28,29^

**Figure 3:**
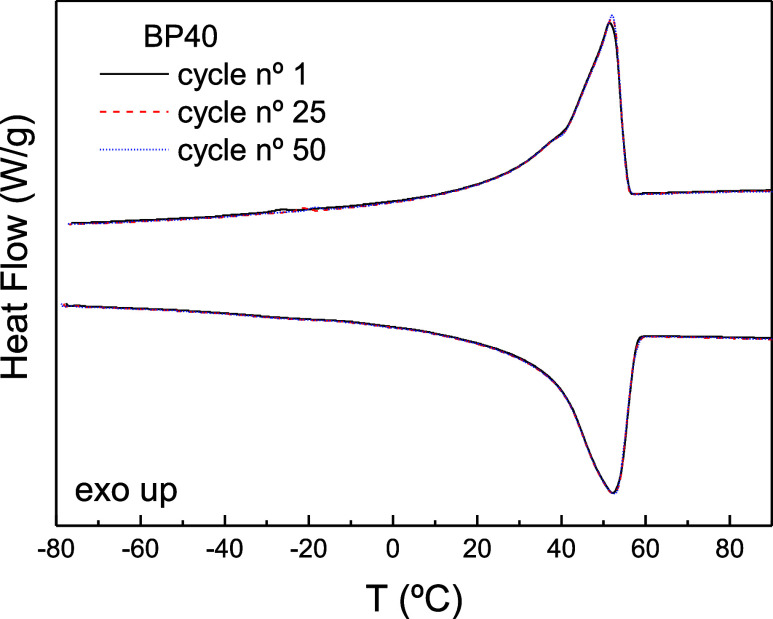
DSC spectra for the BP40 sample after thermal cycling.

In addition to that, the materials proposed here must exhibit suitable thermal storage capacity after a long-term utility period and be able to resist cycling of repeated melting and crystallization cycles. To that end, a thermal cycling test was conducted on a selected blend (BP40) to study its thermal reliability. The heating/cooling DSC procedure was repeated 50 times, and the results for cycle n° 1, 25, and 50 are portrayed in Figure 3. Interestingly, negligible changes in the melting and crystallization curves are noticed on the BP40 binder after thermal cycling, which results in nearly coincident values of characteristic temperatures and enthalpies. Therefore, these results indicate that paraffin wax/bitumen blends can maintain good thermal cycling reliability after a long-term utility period.

**Figure 4:**
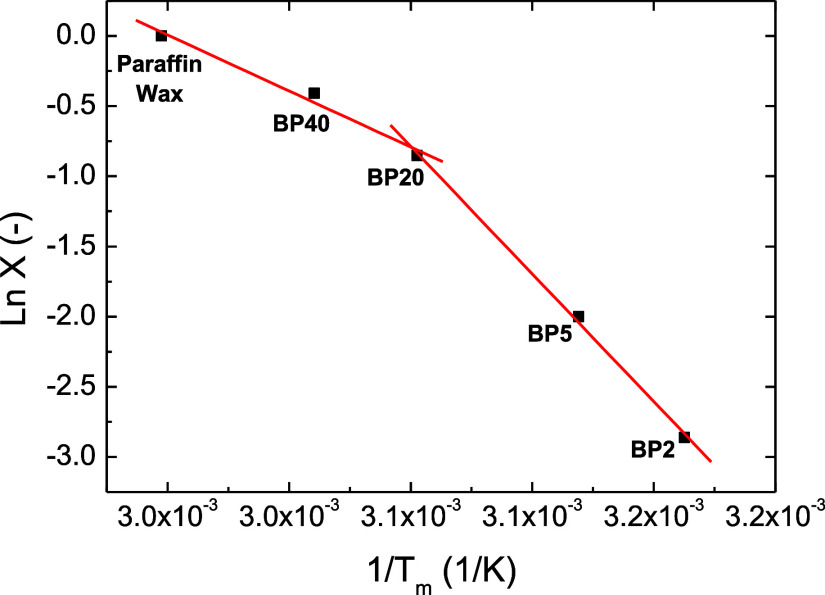
Hildebrand plot of paraffin wax/bituminous blends.

DSC results can also be combined according to Hildebrand eq (eq 1) proving information about the miscibility
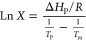
1where “*X*” is the paraffin wax molar fraction, which is calculated from the paraffin wax mass ratio and the molecular weight of both parent components: 370 g/mol for paraffin wax^27^ and a number-average molecular weight of 1100 g/mol for bitumen.^30^ Thus, “*X*” values of 1, 0.66, 0.42, 0.14, and 0.06 are calculated for pristine paraffin wax, BP40, BP20, BP5, and BP2, respectively; Δ*H*_P_ and *T*_P_ are phase change enthalpy (209 J/g) and melting temperature (60.4 °C), respectively, for pristine paraffin wax; *T*_m_ is the melting temperature for the different samples (all of them gathered in Table 1); and *R* is the universal gas constant. As expected from the high crystallinity values in Table 1, it can be stated that paraffin wax and bitumen are not fully miscible in the whole range of paraffin concentration studied, as concluded by the nonlinearity of Ln *X* vs 1/*T*_m_ in Figure 4.^31^

However, data plotted in Figure 4 could be fitted to two different trend lines with a threshold paraffin concentration of 20 wt %. As will be discussed later, a similar conclusion will be drawn from the ring-and-ball softening temperatures conducted on the paraffin wax/bituminous blends, which is indicative of the development of microstructural changes responsible for these different trends.

**Figure 5:**
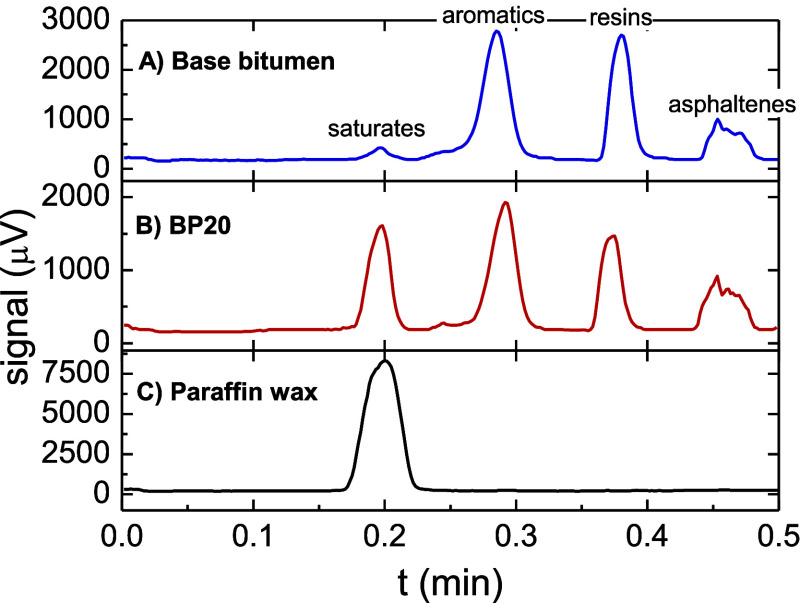
TLC/FID chromatograms for: (A) base bitumen, (B) a selected bituminous blend (BP20), and (C) pure paraffin wax.

Once the results from DSC indicate that it is possible to prepare binary wax/paraffin wax blends with a high crystallinity degree (or a large capacity to store thermal energy), their chemical composition, in terms of “SARAs” fraction, will be studied. Figure 5 displays chromatograms obtained by TLC/FID for base bitumen, a selected wax paraffin-bitumen blend (BP20), and pure paraffin wax. It can be observed that four peaks (which stand for the so-called “SARAs” fractions) corresponding to saturates (S), aromatics (A), resins (R), and asphaltenes (As), respectively, are presented by both base bitumen and BP20 blend. On the other hand, paraffin wax is fully eluted by the solvent used to separate the saturates fraction (heptane), and consequently, paraffin wax displays a single signal located at the bitumen saturate peak position.

**Figure 6:**
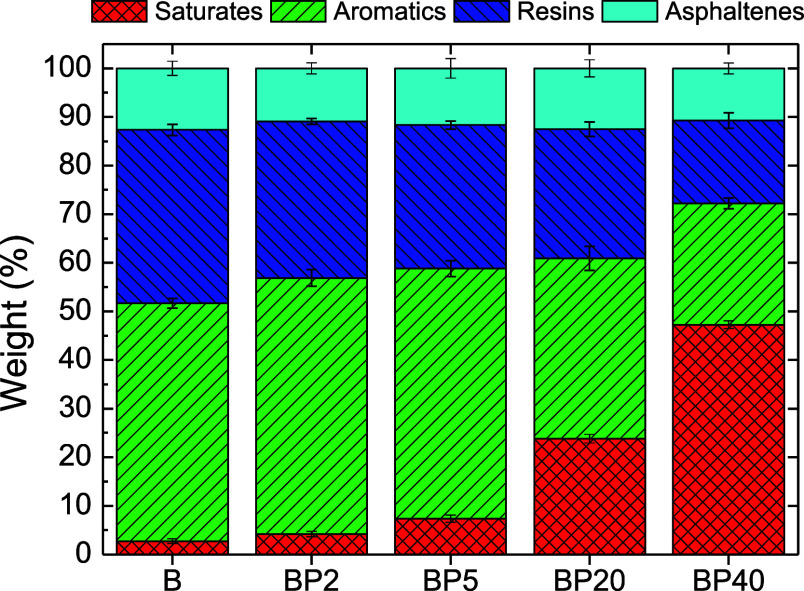
Bitumen “SARAs” fractions for base bitumen and its corresponding paraffin wax/bituminous blends.

Graphic integration of the chromatogram peaks allows us to quantify the different “SARAs” fractions. Figure 6 gathers the weight percentage of every fraction for the base bitumen and its corresponding paraffin wax/bituminous blends.

With increasing concentrations of paraffin wax added to base bitumen, three different effects are noticed: (i) saturate fractions increase significantly, (ii) aromatics and resins decrease progressively, and (iii) asphaltenes remain almost constant. It has been reported that the asphaltene fraction is the most sensitive to change after chemical bitumen modification;^32−34^ however, the chromatographic results indicated that paraffin wax addition significantly affects other families of SARAs compounds since the initial relative proportion of asphaltenes, aromatics, and resins is not conserved. Therefore, it is expected a notable modification of the colloidal arrangement of bitumen compounds. On the other hand, since saturate compounds of bitumen are chemically similar to paraffin wax, and therefore, are eluted together, saturate content follows an expected evolution. Thus, the saturate fraction of the base bitumen (ca. 2.7 wt %) steadily increases with paraffin addition up to ca. 4.2, 7.4, 23.6, and 47.1 wt % for BP2, BP5, BP20, and BP40 blends, respectively. This outcome points out a similar polarity and confirms the mentioned partial miscibility of the saturates with paraffin crystals.

**Figure 7:**
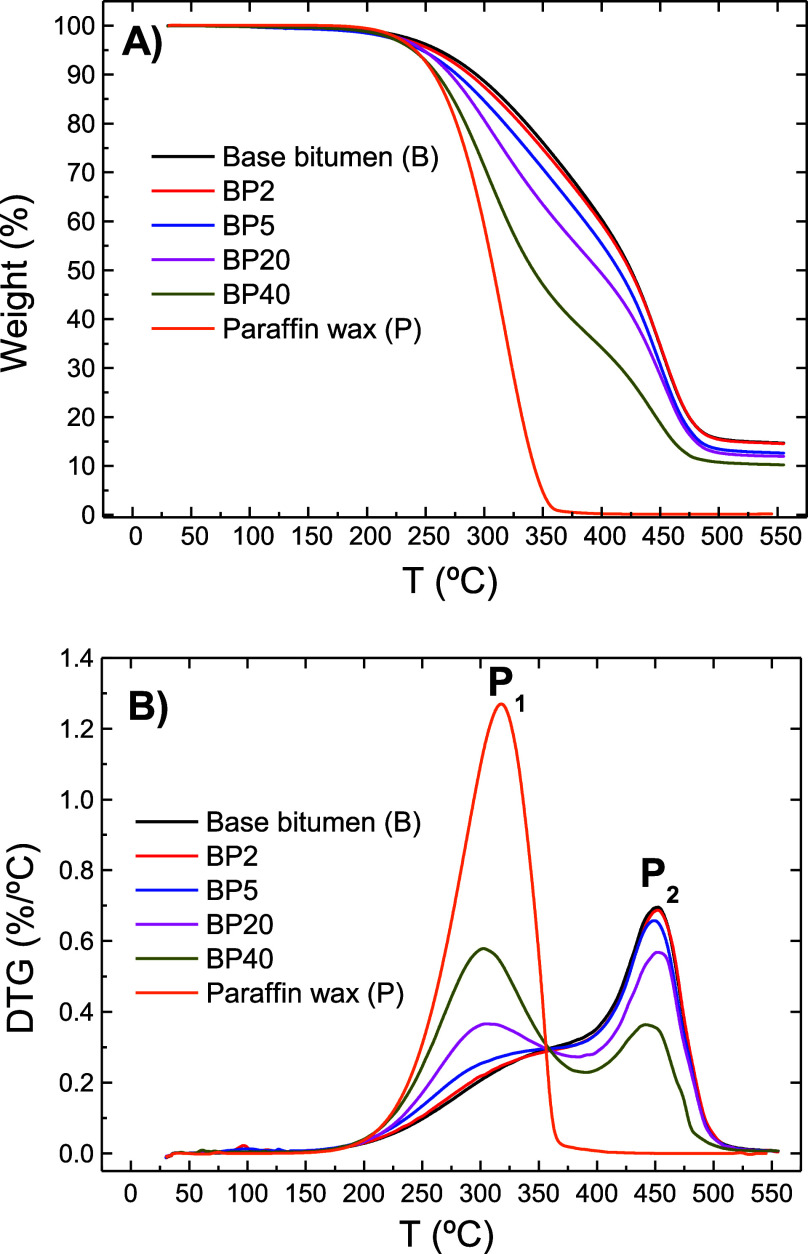
Weight loss (A) and its derivative (B), between 30 and 550 °C and under a N_2_ atmosphere, for base bitumen, pure paraffin wax, and their corresponding paraffin wax/bituminous blends.

The thermal stability of the paraffin wax/bitumen blends was studied by employing TGA. Figure 7 shows the weight loss and its derivative (DTG) for base bitumen, paraffin wax, and their corresponding blends. In addition, Table 2 gathers the characteristic parameters of these curves, namely, the temperature for a weight loss of 2% (*T*_2%_), the temperature at which the thermal decomposition rate is maximum (*T*_max_), and the percentage of nondegraded residue at 550 °C. As may be observed, paraffin wax displays a typical one single degradation stage, with the maximum rate (*P*_1_) located at 317.4 °C showing virtually no char residues, pointing out that paraffin undergoes a simple evaporation process. Base bitumen displays a loss process in a wider temperature range formed by two overlapped peaks with its maximum (*P*_2_) located at 450.8 °C, which involves the decomposition/volatilization of chemical compounds with very different molecular weights. In addition, bitumen decomposition leads to a char content of 14.7% which is a typical response of the thermal degradation of polycyclic condensed aromatic hydrocarbons compounds. As for paraffin wax/bituminous blends, their decomposition occurs in two stages at similar temperatures to those noticed on their parent components. As seen in Figure 7, the magnitude of these degradation stages and the percentage of nondegraded residue at 550 °C are dependent on the blends’ composition.

**Table 2:** Characteristic Parameters Obtained from TGA Measurements for Base Bitumen, Pure Paraffin Wax, and Their Corresponding Paraffin Wax/Bituminous Blends

		*T*_max_ (°C)		
	*T*_2%_(°C)	*P*_1_	*P*_2_	residue_550 °C_ (wt %)
B	261.0 ± 1.5		450.8 ± 1.0	14.68 ± 0.5
BP2	255.6 ± 1.1		451.0 ± 1.2	14.58 ± 0.7
BP5	246.8 ± 1.8		448.6 ± 0.8	12.64 ± 1.0
BP20	248.1 ± 2.1	302.6 ± 0.9	451.7 ± 1.5	11.95 ± 0.8
BP40	239.5 ± 1.8	304.7 ± 1.2	443.1 ± 1.3	10.22 ± 0.9
P	240.9 ± 1.4	317.4 ± 1.1		0.16 ± 0.1

From a performance point of view, it is noteworthy that all binders present high thermal stability since their initial decomposition temperatures are clearly higher than the specific temperature range for low-temperature solar energy collection (40–80 °C),^26,35^ even allowing them to be used at the high temperatures required in the asphalt paving industry.^36^

**Figure 8:**
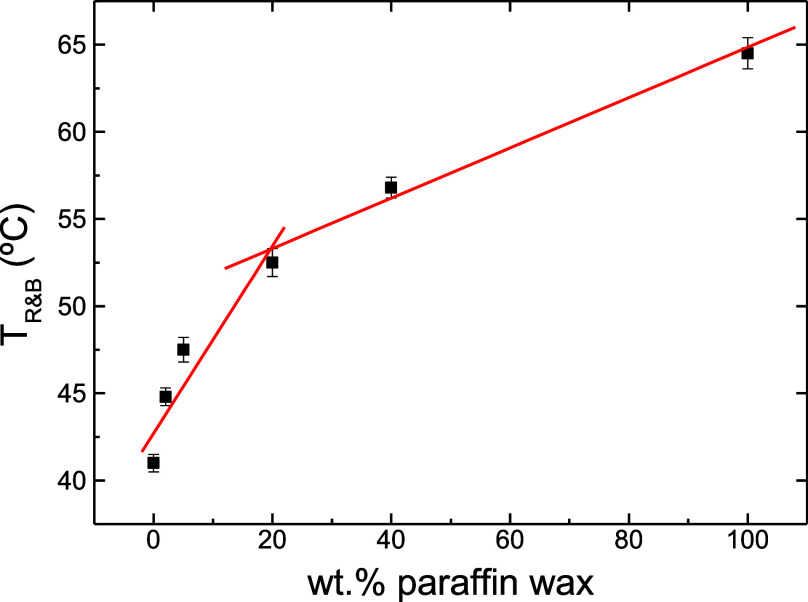
Effect of the paraffin wax concentration on the ring-and-ball softening temperature.

A common bitumen characterization parameter that provides valuable structural information for the high-temperature performance of the bituminous binders is the ring-and-ball softening temperature. Figure 8 shows the evolution of this parameter with the paraffin wax concentration.

The addition of paraffin wax produces a hardening of the base bitumen, as deduced from the increase in *T*_R&B_ in Figure 8. Thus, this parameter is strongly correlated to the melting process of the crystalline structure and points out that the major softening occurs at a temperature just above the DSC melting peak (see values of *T*_m_ in Table 1). Again, similar to what was observed in the Hildebrand plot, a change in the slope at a 20 wt % paraffin wax content is noticed. The different evolution of the *T*_R&B_ at low and high paraffin wax concentration could be attributed to microstructural changes caused by a phase inversion at around 20 wt % paraffin wax content. Thus, at low paraffin wax concentration, the dispersed paraffin wax added to bitumen can act as a reinforcing agent for the continuous bitumen matrix, significantly increasing *T*_R&B_ values until the melting transition is reached in the heating test. Above this critical concentration of the phase inversion, a continuous paraffin-rich phase is developed which controls the thermomechanical response of the binder.

**Figure 9:**
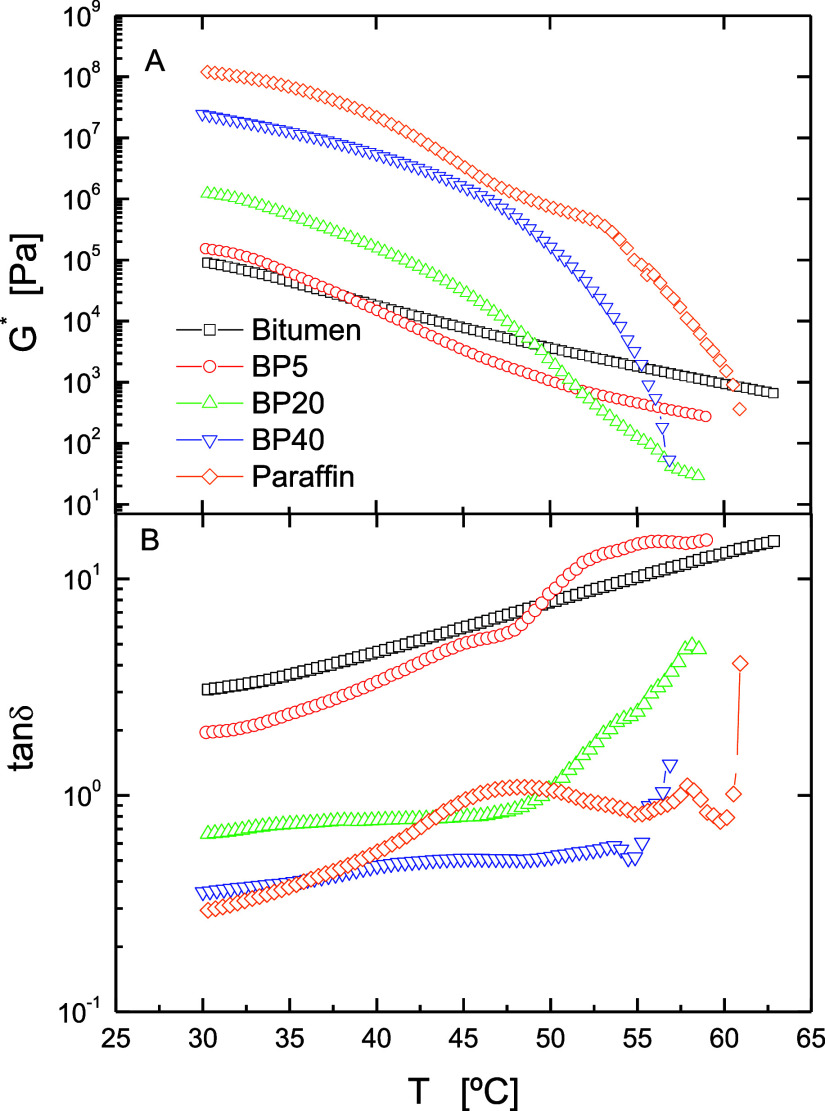
Evolution of (A) complex shear modulus and (B) loss tangent with testing temperature for base bitumen, paraffin wax, and their blends.

Evaluation of the rheological properties is one of the most important aspects to be considered for applications such as paving or roofing, in which bitumen acts as a supporting engineering material. Thus, Figure 9 confirms the structural change with concentration and shows the evolution with temperature of both complex shear modulus (stiffness and overall resistance to deformation), G*, and loss tangent (inversely proportional to the elasticity of the binder), tan δ, for base bitumen, paraffin wax, and their corresponding blends. Neat bitumen presents a monotonous decrease in G*, with a predominantly viscous character (tan δ > 1), which is more apparent as temperature rises. With regard to paraffin wax, this sample shows the highest values of G* in the testing temperature range, with a predominant elastic character (tan δ < 1), but displays a more complex evolution with temperature. As may be seen, G* of paraffin wax begins to decrease and, next, it undergoes two flattening in its slope prior to the melting process (60.4 °C), which are reflected in two maxima in tan δ located at ca. 48 and 58 °C, respectively. At higher temperatures, and as a result of the melting of the crystalline structures, tan δ presents a sharp rise and the terminal region of the mechanical spectrum is reached. This result can be explained by the fact that, in the testing temperature range, paraffin wax may present several layered plastic crystalline mesophases that lead to solid–solid transitions.^37−39^

The thermomechanical behavior of blends depends on which compound forms the continuous phase and controls the rheological behavior. Then, BP5 shows the expected behavior of a bitumen-rich continuous phase, where dispersed paraffins melt during heating. By contrast, the BP40 sample exhibits a behavior closer to that shown by paraffin, which points out that a continuous paraffin-rich phase has developed. Thus, a predominately elastic behavior is pointed out, and the flattening processes are weakly noticed in tan δ, before its melting point (54.9 °C) at lower temperatures than those for paraffin wax. Finally, BP20 displays an intermediate behavior attributed to the mentioned microstructural change and phase inversion.

### 3.2 Temperature Regulation Performance

**Figure 10:**
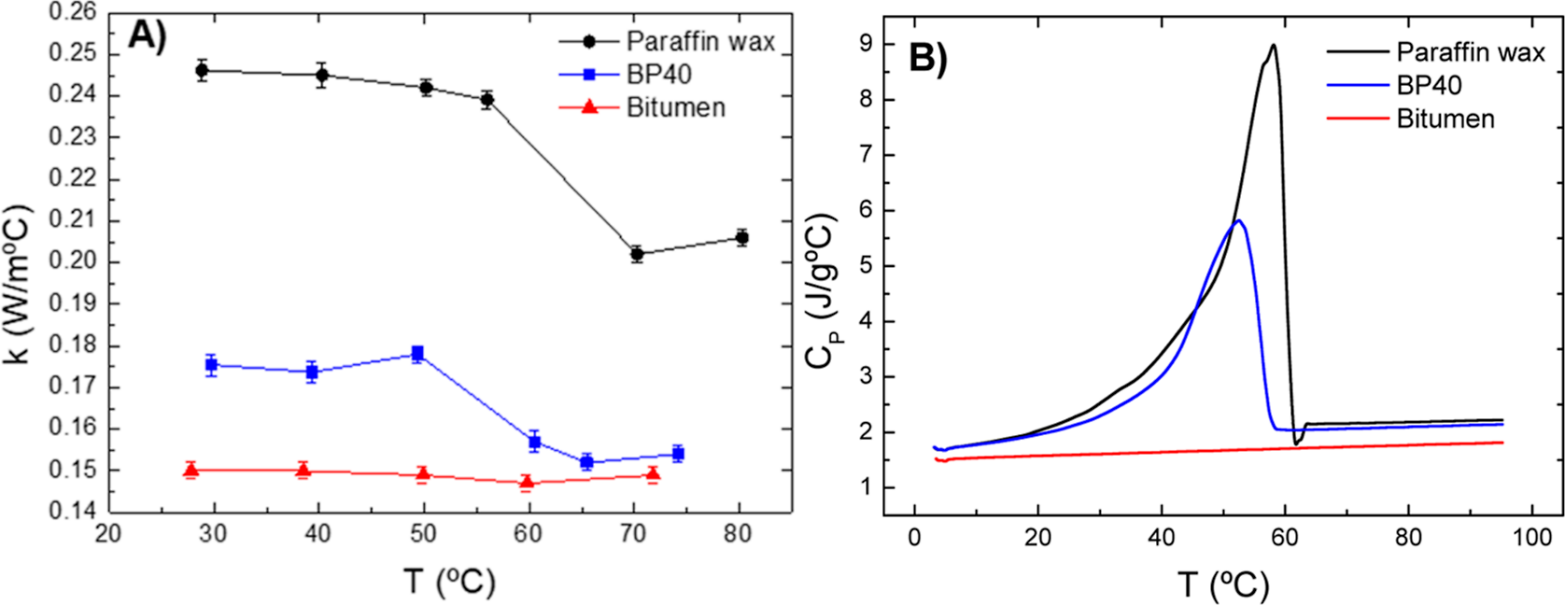
Evolution of thermal conductivity (A) and specific heat capacity (B) with testing temperature for base bitumen, paraffin wax, and a selected blend (BP40).

An efficient PCM should exhibit not only a high latent heat but also a combination of favorable thermal properties that enhances the thermal energy storage. To study this, Figure 10 shows the thermal conductivity and specific heat capacity as a function of testing temperature for base bitumen, paraffin wax, and the selected BP40 blend. On the one hand, thermal conductivities hardly change for base bitumen, displaying the lowest values. However, paraffin wax shows higher and constant conductivities until the melting process begins, which is noticed as an abrupt drop of values. A similar trend is also observed for the BP40 blend but with a less pronounced drop occurring at lower temperature (in agreement with its lower melting point).

On the other hand, the specific heat capacity of bitumen slightly increases with testing temperature, and paraffin wax presents a significant asymmetric peak centered at ca. 60 °C due to the heat absorption process occurring during the phase change. Interestingly, the BP40 blend presents higher specific heat capacities than base bitumen in the whole testing temperature window, especially between 40 and 60 °C, the range in which the thermoregulation effect for solar energy heating applications is considered.^36^ In addition to that, the BP40 blend shows higher thermal conductivities than base bitumen, a fact that reduces the time for the heat charging/discharging processes and, thereby, enhances the efficiency of the thermal energy storage.

**Figure 11:**
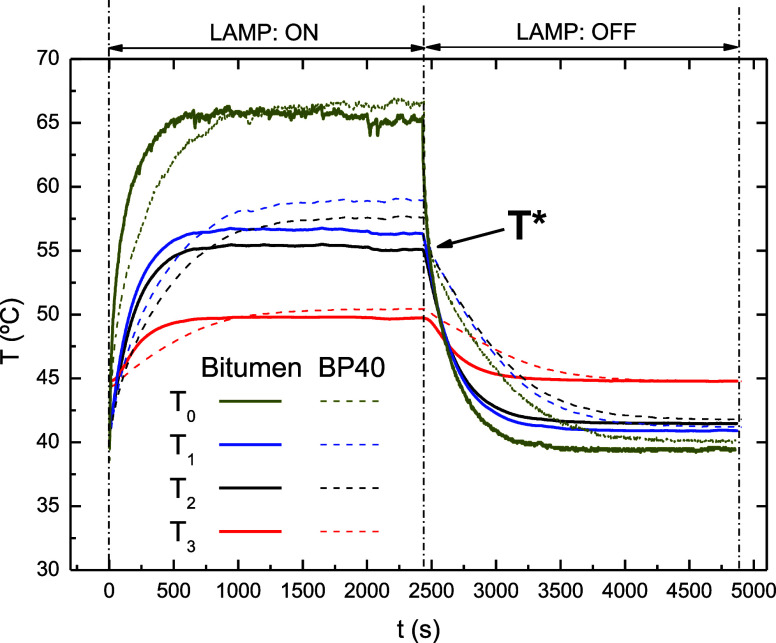
Evolution of the different temperature sensors (see Figure 1) with time during the temperature regulation test for base bitumen and the selected blend (BP40).

Aiming to study the temperature regulation performance of the BP40 blend compared to base bitumen, and according to the experimental setup described in subsection 2.3.7, the following protocol was conducted: (i) first, samples were subjected to the action of the solar lamp using a constant radiant flux density (q* = 1460 W/m^2^ at the sample center), and (ii) subsequently, the lamp was turned off. To ensure that all paraffin wax in the BP40 sample melts, the sink temperature (*T*_S_) was set at 46.5 °C, which is close to the onset of its melting temperature. Thus, by selecting *T*_ambient_ = 21 °C, *T*_S_ = 46.5 °C, and q* = 1460 W/m^2^, the sample temperatures at the bottom and top take values of ca. 46.5 and 65 °C, respectively. Figure 11 displays the evolution of the different temperature sensors (Figure 1) with time for both samples.

During the first stage (marked as “LAMP: ON” in Figure 10), the irradiation on the top surface of the sample is partially absorbed (and heat is conducted through the material), and the rest is reflected, emitted as heat radiation and lost by free convection.^40^ Typical patterns of recorded temperatures show a rapid increase of top surface temperature (*T*_0_) until equilibrium (or steady state) is reached, followed by the other temperatures, with lower values as the distance from the top (or depth) is higher (i.e., in equilibrium *T*_0_ > *T*_1_ > *T*_2_ > *T*_3_). The thermoregulation ability of the BP40 sample is clearly noticed by comparing their heating curves with those for base bitumen. Thus, the heat absorption process that occurs during the melting of the crystalline structures dampens the sample temperature, which is reflected in lower initial heating rates compared to base bitumen.

**Figure 12:**
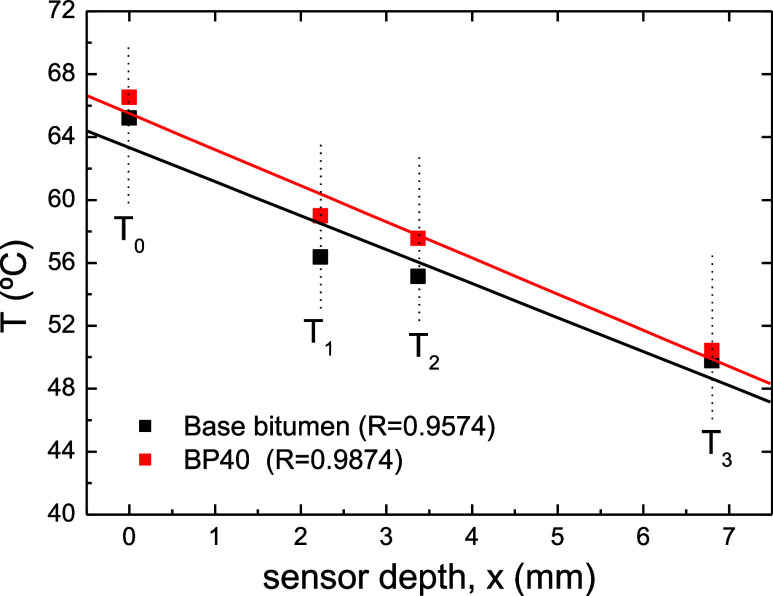
Steady-state temperatures recorded in the first stage of the temperature regulation test as a function of sensor depth for base bitumen and the selected blend (BP40).

The steady-state temperatures, which are recorded at the end of this first stage, will be used to calculate a local heat flux transferred by a conduction mechanism at the sample center, *q*_c_, by means of the Fourier’s law (eq 2)
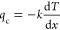
2where *k* is the material thermal conductivity and *x* is related to the depth of each temperature sensor. Figure 12 shows the steady-state temperatures reached by both samples as a function of the sensor depth.

Slopes of the linear fitting from Figure 12 (this is, −*q*_c_/*k*) may be used to obtain local heat flux conducted through the sample center (*q*_c_), which may be compared with the solar irradiation flux at the sample center supplied by the lamp (q* = 1460 W/m^2^) to assess the material capability of solar heat absorption, *q*_abs_ (eq. 3)
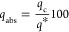
3

Thus, under the selected conditions, solar heat absorption capability increased from 22.2% for base bitumen to an average value of 26.0% for the BP40 sample (which results from considering an average thermal conductivity). Although calculated absorptions might be affected by selected test conditions (e.g., ambient temperature, sink temperature, and solar irradiation flux), these findings are of interest for solar energy applications, since they would suggest an enhanced behavior for the BP40 sample under incident solar energy.

**Figure 13:**
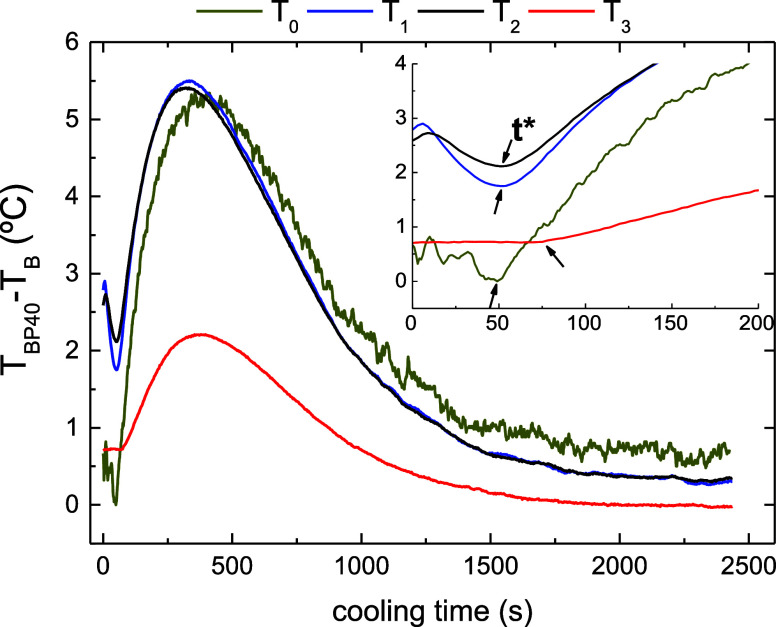
Temperature differences between base bitumen (*T*_B_) and BP40 (*T*_BP40_) samples as a function of the time elapsed after switching off the lamp.

Finally, the second stage of the temperature regulation test (marked as “LAMP: OFF” in Figure 11) can also provide valuable information. As expected, just after the lamp is turned off, there is a decrease in each temperature until equilibrium is reached again. In this case, the thermoregulation effect is noticed on the BP40 sample by the higher temperatures obtained during the cooling process and the lower cooling rates. It is worth noting that this thermoregulation effect begins at a similar temperature of approximately 53 °C (marked as T* in Figure 11) for those sensors located closer to the sample surface (*T*_0_, *T*_1_, and *T*_2_). Then, this temperature matches with the onset of the exothermic crystallization process in the cooling DSC scan of BP40 (Figure 2) and therefore confirms that this dampening of the temperature drop is due to the heat released in the course of the crystallization of paraffin wax. This can be clearly visualized in Figure 13, in which temperature differences between both samples are plotted versus the time elapsed after the lamp.

Thus, after ca. 50 s without solar irradiation (time marked as t* in Figure 13), a continuous increase in *T*_BP40_–*T*_B_ signals related to the initiation of thermoregulation effect is noticed on *T*_0_, *T*_1_, and *T*_2_ sensors, whereas this time increased up to ca. 75 s for the sensor placed deeper (*T*_3_). Afterward, temperature differences reach a maximum and, finally, tend to level off. The data displayed in Figure 13 can be used to calculate the latent heat thermoregulation index (LHTI), according to that reported by Ma et al.,^13^ as follows
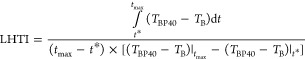
4where t* is the time when thermoregulation effect begins and *t*_max_ is the time at which the maximum temperature difference is reached. Thus,  indicates the thermal regulation ability of a PCM with the accumulation of temperature difference in a certain time range, and LHTI reflects the efficiency of latent heat thermoregulation.^13^ Interestingly, similar LHTI values of 1.17, 1.21, and 1.14 were calculated for *T*_1_, *T*_2_, and *T*_3_ sensors, respectively, a result that highlights the homogeneous distribution of paraffin wax in the BP40 sample, as well as its proper stability during the thermoregulation test. Although the value of this index depends on the temperature cooling rate used in the test,^14^ LHTI values here obtained are higher than those obtained after adding 6 wt % microencapsulated PEG-2000 to bitumen (LHTI value of ca. 0.7 at a fast cooling rate of 10 °C/min)^14^ or 7 wt % polyurethane solid–solid PCM (LHTI value of ca. 0.1 at 2 °C/min).^41^ Therefore, the selected paraffin wax/bituminous blend proposed here shows great potential for its use for solar energy storage applications with thermoregulation purposes.

## 4 Concluding Remarks

The miscibility, thermal stability, thermomechanical properties, and temperature regulation performance of paraffin wax/bitumen blends were evaluated. A partial miscibility of both components was observed, but they form independent domains at a microscale level, which allows to obtain blends with high crystallinity and, therefore, with a large latent heat to be used for solar energy storage applications. These blends present a suitable thermal stability, and their thermomechanical properties (evaluated by ring-and-ball softening point and rheological response) are strongly dependent on composition, developed microstructure, and temperature. At low paraffin content (<20 wt % paraffin), the disperse paraffin-rich phase acts as a filler that reinforces the continuous bitumen matrix until the melting transition is reached in heating tests. Above the critical concentration of the phase inversion, the continuous paraffin-rich phase controls the thermomechanical response. Interestingly, oscillatory-shear temperature sweep tests conducted on a blend containing 40 wt % wax (BP40) displays enhanced binder elastic properties with lower thermal susceptibility compared to base bitumen. In addition to that, BP40 also displays enhanced thermal properties (thermal conductivity and specific heat capacity), which makes it a suitable material to be used in solar energy storage applications. To that end, the temperature regulation performance of the BP40 blend compared to base bitumen was determined by subjecting samples to simulated solar irradiation at a constant radiant flux density of 1460 W/m^2^. Results revealed that solar heat absorption capability increased from 22.2% for base bitumen to 26.0% for the BP40 sample, which would result in a better optimization of incident solar energy. Finally, compared to other microencapsulated PCMs used with bitumen, the LHTI calculated for the BP40 sample points out the great potential of these materials to formulate form-stable products for solar thermal energy storage applications with thermoregulation purposes.
